# Inhibition of P2X7 Purinergic Receptor Ameliorates Fibromyalgia Syndrome by Suppressing NLRP3 Pathway

**DOI:** 10.3390/ijms22126471

**Published:** 2021-06-16

**Authors:** Ramona D’Amico, Roberta Fusco, Rosalba Siracusa, Daniela Impellizzeri, Alessio Filippo Peritore, Enrico Gugliandolo, Livia Interdonato, Andrea Maria Sforza, Rosalia Crupi, Salvatore Cuzzocrea, Tiziana Genovese, Marika Cordaro, Rosanna Di Paola

**Affiliations:** 1 Department of Chemical, Biological, Pharmaceutical and Environmental Sciences, University of Messina, 98166 Messina, Italy; rdamico@unime.it (R.D.); rfusco@unime.it (R.F.); rsiracusa@unime.it (R.S.); dimpellizzeri@unime.it (D.I.); aperitore@unime.it (A.F.P.); livia.interdonato@yahoo.it (L.I.); or andrea.sforza93@yahoo.com (A.M.S.); dipaolar@unime.it (R.D.P.); 2 Department of Veterinary Sciences, University of Messina, 98168 Messina, Italy; egugliandolo@unime.it (E.G.); rcrupi@unime.it (R.C.); 3 Department of Biomedical, Dental and Morphological and Functional Imaging, University of Messina, via Consolare Valeria, 98125 Messina, Italy; cordarom@unime.it

**Keywords:** fibromyalgia, P2X7 receptor, NLRP3 inflammasome, neuroinflammation

## Abstract

Fibromyalgia is a chronic condition characterized by persistent widespread pain that significantly reduces quality of life in patients. The purinergic P2X7 receptor (P2X7R) seems to be involved in different pain states and neuroinflammation. The purpose of this study is to investigate the positive effects of P2X7R inhibition by the antagonist Brilliant Blue G (BBG) in a rat model of reserpine-induced fibromyalgia. Sprague–Dawley male rats were injected with 1 mg/kg of reserpine for three consecutive days. Later, animals were administered BBG (50 mg/kg) intraperitoneally for seven days. Reserpine injections induced a significant increase in pain pro-inflammatory mediators as well as a significant increase in neuroinflammation. Chronic pain, in turn, led to depressive-like symptoms and reduced neurogenesis. Blockage of P2X7R by BBG administrations is able to attenuate the behavioral deficits, pain mediators and microglial activation induced by reserpine injection. Additionally, BBG prevents NLRP3 inflammasome activation and consequently the release of active interleukin (IL)-1 and IL-18, involved in the activation of nociceptors. In conclusion, these results suggest that inhibition of P2X7R should be further investigated to develop a potential approach for the management of fibromyalgia.

## 1. Introduction

Fibromyalgia (FM) is a multifactorial chronic syndrome, characterized by chronic pain and tenderness (generalized allodynia/hyperalgesia) accompanied by other somatic and psychological symptoms including fatigue, sleep and mood disturbances and cognitive dysfunction [1,2]. Despite significant developments in understanding its pathophysiology, the etiology of FM is still unknown. Depression and anxiety disorders are highly comorbid with chronic pain, maybe due to the stress effects of the condition [3,4], and both are associated with decreases in hippocampal neurogenesis, that may worsen or exacerbate FM symptomatology [5,6]. While the cause for such changes is currently unknown, there is evidence that FM is associated with disturbances in pain processing by the central nervous system (CNS) [7]. Both hyperalgesia and allodynia reflect an enhanced CNS processing of painful stimuli that is characteristic of central sensitization [8], which develops as a result of the plasticity of neuronal synapses [9]. The immune system and neuroinflammation play a crucial role in sensitization of CNS, including activation of nociceptors. The latter are triggered by proinflammatory mediators such as adenosine triphosphate (ATP) or interleukin-1β (IL-1β), resulting in a heightened sensitivity to pain and contributing to the symptomatology of FM [10].

Recent evidence suggests that the purine molecule ATP plays a key role in the regulation of nociceptive transmission [11]. Notably, the P2X7 receptor (P2X7R) is an ATP-gated ion channel that plays an important role in the inflammatory response and in different pain states [12,13]. P2X7R is overactivated due to ATP release, resulting in anion imbalance and triggering of microglia, that additionally exacerbate cell damage [14,15]. Molecules targeting the specific receptors for ATP have been proven to be effective in modulating different pain conditions [16]. P2X7R activation is involved in several signaling pathways [17]; in particular, the NLRP3 inflammasome pathway [18]. It is well known that the activation of NLRP3 leads to release of IL-1β, as well as release of active IL-18, which has been discovered to mediate painful conditions including neuropathic pain and cancer-induced bone pain [18,19,20]. Importantly, IL-1β can act as an inflammatory mediator serving to upregulate downstream cytokines and directly activate nociceptive neurons [21]. Thus, targeting inflammasomes or, further upstream, P2X7R may more efficiently modulate activation of IL-1β and IL-18 and therefore more effectively inhibit downstream cytokine cascades compared with traditional therapies aimed at countering the effect of individual cytokines. On that note, it has been demonstrated that Brilliant Blue G (BBG) is a selective and non-competitive P2X7R antagonist with good blood–brain barrier permeability [22]. BBG serves a neuroprotective role in several experimental models by attenuating microglial activation, reducing thermal hyperalgesia or promoting motor function recovery [23,24,25].

Based on the above considerations, we investigated the possible protective effect of blockage of P2X7R by BBG in a rat model of reserpine-induced fibromyalgia. Indeed, it has been widely demonstrated that reserpine injections cause a dysfunction of the control mediated by biogenic amines in the CNS that would lead to a disease condition that mimics FM.

## 2. Results

### 2.1. Effects of Inhibition of P2X7R by BBG on Mast Cell Degranulation and Pain-Related Pro-Inflammatory Mediators in Spinal Cord

**Figure 1:**
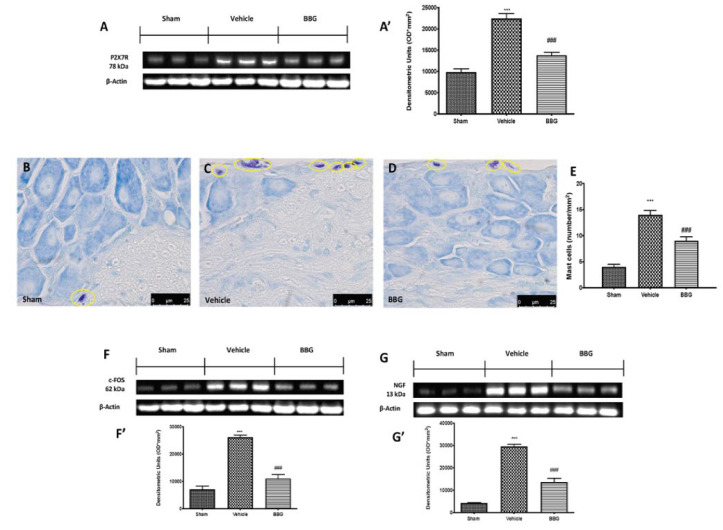
Western blot and respective quantification of P2X7R (**A**,**A’**), c-FOS (**F**,**F’**) and NGF (**G**,**G’**). Evaluation of mast cell degranulation: sham (**B**), vehicle (**C**), BBG (**D**). Mast cell density (mast cell numbers per unit area of tissue; (**E**). Data are representative of at least three independent experiments and are expressed as mean ± SEM from N = 6 rats/group. *** *p* < 0.001 vs. sham; ### *p* < 0.001 vs. vehicle.

At the end of the experiment, we first observed whether daily BBG administrations were actually able to reduce P2X7R protein expression in the spinal cord. Western blot analysis confirmed that BBG significantly decreased P2X7R expression, compared to the vehicle group. Sham animals showed a basal expression of P2X7R (Figure 1A,A’). 

To investigate the activation of mast cells, which are known to act on nociceptor and sensory neurons, we performed toluidine blue staining on spinal cord sections. Seven days after the last reserpine induction, the number of mast cells was significantly increased in vehicle groups (Figure 1C,E) compared to the sham group (Figure 1B,E). P2X7R inhibition by BBG reduced the number of intact and degranulated mast cells in the dorsal horn of the spinal cord (Figure 1D,E). As c-FOS are closely associated with mast cell degranulation and linked to algesic stimuli, the expression of this marker was evaluated by Western blot analysis on spinal cord tissues. Seven days after the last reserpine induction, a significant increase in c-FOS expression in the vehicle group was found. On the contrary, intraperitoneal administrations of BBG significantly reduced the expression of c-FOS, as shown in Figure 1F,F’. Additionally, several pieces of evidence suggest that nerve growth factor (NGF) is triggered by mast cell degranulation and seems to be associated with hyperalgesia. In that regard, NGF expression was evaluated by Western blot analysis. An increase in NGF expression was found in the spinal cord collected from vehicle rats compared to controls. Daily BBG treatment was able to downregulate NGF expression (Figure 1G,G’).

### 2.2. Effects of Inhibition of P2X7R by BBG on Astrocytes and Microglia Activation 

To study the involvement of neuroinflammation in FM, we evaluated expression of glial fibrillary acidic protein (GFAP) and ionized calcium binding adaptor molecule 1 (Iba1), astrocytes and microglia activation markers, respectively.

**Figure 2:**
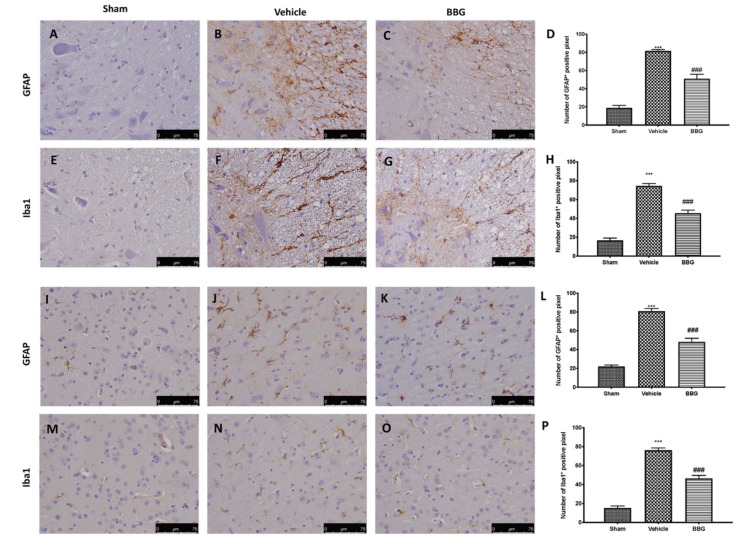
Immunohistochemical evaluation in spinal cord for GFAP expression: Sham (**A**), Vehicle (**B**), BBG (**C**) and Iba1 expression: Sham (**E**), Vehicle (**F**), BBG (**G**). Graphical quantification of GFAP (**D**) and Iba1 expression (**H**) in spinal cord. Immunohistochemical evaluation in hypothalamus region for GFAP expression: Sham (**I**), Vehicle (**J**), BBG (**K**) and Iba1 expression: Sham (**M**), Vehicle (**N**), BBG (**O**). Graphical quantification of GFAP (**L**) and Iba1 expression (**P**) in hypothalamus region. Data are representative of at least three independent experiments and are expressed as mean ± SEM from N = 6 rats/group. *** *p* < 0.001 vs. sham; ### *p* < 0.001 vs. vehicle.

Immunohistochemical analysis revealed a significant increase in GFAP expression in spinal cord sections in FM animals (Figure 2B,D), compared to sham group (Figure 2A,D). Inhibition of P2X7R by BBG significantly reduce the number of positive cells for GFAP (Figure 2C,D), compared to the vehicle group. Similar results were observed for Iba1, the expression of which was increased in the vehicle group (Figure 2F,H), compared to sham group (Figure 2E,H). BBG treatment was able to decrease activation of microglia as demonstrated by downregulation of Iba-1 expression (Figure 2G,H), compared to vehicle group. 

At the same way, we detected astrocyte and microglial cell activation also in brain section, in particular in hypothalamus region, as it has recently been proposed to be involved in neuroinflammation during FM [26]. A significant increase in the number of positive cells for GFAP and Iba-1 were observed after FM induction (Figure 2J,L,N,P respectively), compared to control (Figure 2I,L,M,P respectively). Administrations of BBG at dose of 50 mg/kg significantly reduced the expression of these markers of neuroinflammation also in hypothalamus area (Figure 2K,L for GFAP and Figure 2O,P for Iba-1). 

### 2.3. Effects of Inhibition of P2X7R by BBG on NLRP3 Inflammasome Pathway in Spinal Cord Samples 

**Figure 3:**
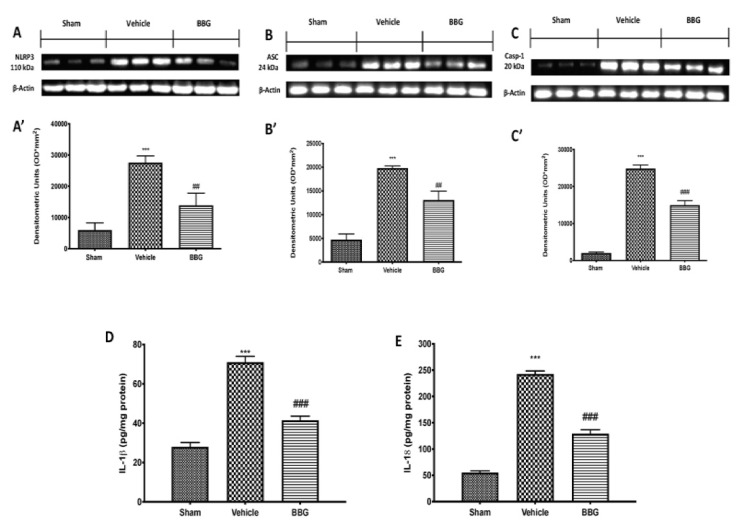
Western blot and respective quantification of NLRP3 (**A**,**A’**), ASC (**B**,**B’**) and Casp-1 (**C**,**C’**) in spinal cord tissue. Cytokines expression: IL-1β (**D**) and IL-18 (**E**). Data are representative of at least three independent experiments and are expressed as mean ± SEM from N = 6 rats/group. *** *p* < 0.001 vs. sham; ## *p* < 0.01 vs. vehicle; ### *p* < 0.001 vs. vehicle.

To better explore which signaling pathway could be involved in the neuroinflammatory response in FM, we performed Western blot analyses for the NLRP3 inflammasome pathway. The results obtained show an important increase in NLRP3 expression in spinal cord samples collected from the vehicle group, while NLRP3 levels were significantly reduced in the BBG group (Figure 3A,A’). Western blot analysis also displayed an upregulation of ASC (Figure 3B,B’) and Caspase 1 (Casp-1, Figure 3C,C’) levels in FM rats, which were significantly reduced after BBG treatment. 

Seven days after the last reserpine injection, IL-1 β (Figure 3D) and IL-18 (Figure 3E) levels were increased in vehicle animals, compared to the sham groups. Inhibition of P2X7R by BBG produced a significant reduction in both pro-inflammatory cytokines’ expressions in spinal cord samples.

### 2.4. Effects of Inhibition of P2X7R by BBG on NLRP3 Inflammasome Pathway in Brain Samples

**Figure 4:**
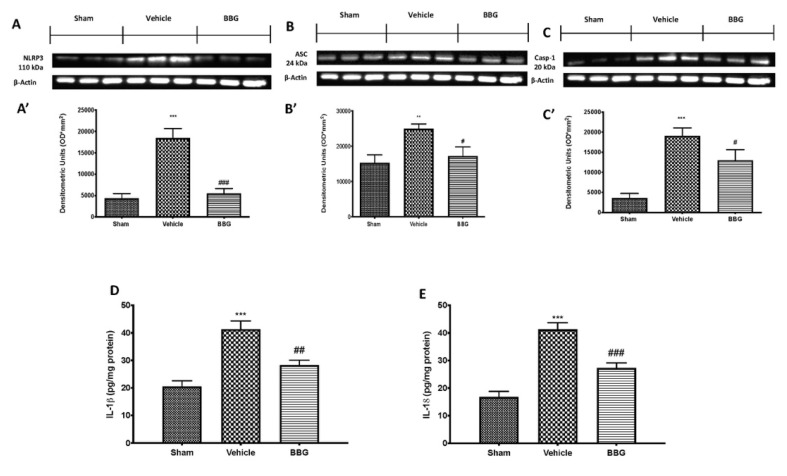
Western blot and respective quantification of NLRP3 (**A**,**A’**), ASC (**B**,**B’**) and Casp-1 (**C**,**C’**) in brain tissue. Cytokines expression: IL-1β (**D**) and IL-18 (**E**). Data are representative of at least three independent experiments and are expressed as mean ± SEM from N = 6 rats/group. ** *p* < 0.01 vs. sham; *** *p* < 0.001 vs. sham; # *p* < 0.05 vs. vehicle; ## *p* < 0.01 vs. vehicle; ### *p* < 0.001 vs. vehicle.

In the same way, BBG administration, targeting its ATPase activity, modulated NLRP3 inflammasome pathway activation in brain tissues. Western blot analysis showed a significant increase in NLRP3 (Figure 4A,A’), ASC (Figure 4B,B’) and Casp-1 (Figure 4C,C’) in the vehicle group compared to sham animals. BBG administration reduced the expression of all components of NLRP3 inflammasome in brain samples.

IL-1β and IL18 levels were also measured in brain tissues. Our results display that intraperitoneal administration of BBG significantly reduced IL-1 β (Figure 4D) and IL-18 (Figure 4E) levels compared to vehicle rats.

### 2.5. Effects of Inhibition of P2X7R by BBG on Mechanical and Thermal Hyperalgesia and Allodynia

**Figure 5:**
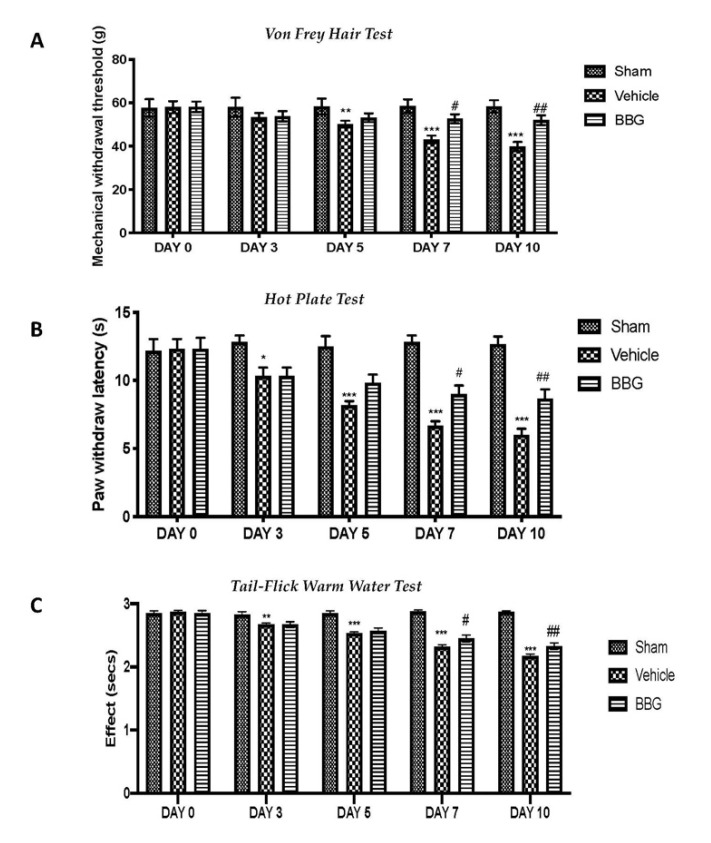
Behavioral analysis: von Frey hair test (**A**), hot plate test (**B**) and tail-flick warm water test (**C**). Data are representative of at least three independent experiments and are expressed as mean ± SEM from N = 6 rats/group. * *p* < 0.05 vs. sham; ** *p* < 0.01 vs. sham; *** *p* < 0.001 vs. sham; # *p* < 0.05 vs. vehicle; ## *p* < 0.01 vs. vehicle.

To investigate whether P2X7R inhibition by BBG was able to reduce pain associated with FM, we subjected the rats to several behavioral tests. No significant differences were observed in mechanical hyperalgesia and allodynia in all animals before the administration of reserpine. Mechanical hyperalgesia was evaluated by von Frey test. Reserpine injection reduced the paw-withdrawal threshold in response to von Frey hair stimulation in vehicle rats, compared to sham groups. Intraperitoneal administrations of BBG significantly increased the paw-withdrawal threshold compared to vehicle group (Figure 5A).

In addition, pain sensitivity was tested by subjecting rats to hot plate (Figure 5B) and tail-flick warm water (Figure 5C) tests. Subcutaneous injection of reserpine produced an increased pain sensitivity in the vehicle group compared to sham rats. Inhibition of P2X7R by BBG treatment attenuated reserpine-induced alterations in thermal hyperalgesia and allodynia compared to vehicle rats.

### 2.6. Effects of Inhibition of P2X7R by BBG on Depressive-Like Behavior

**Figure 6:**
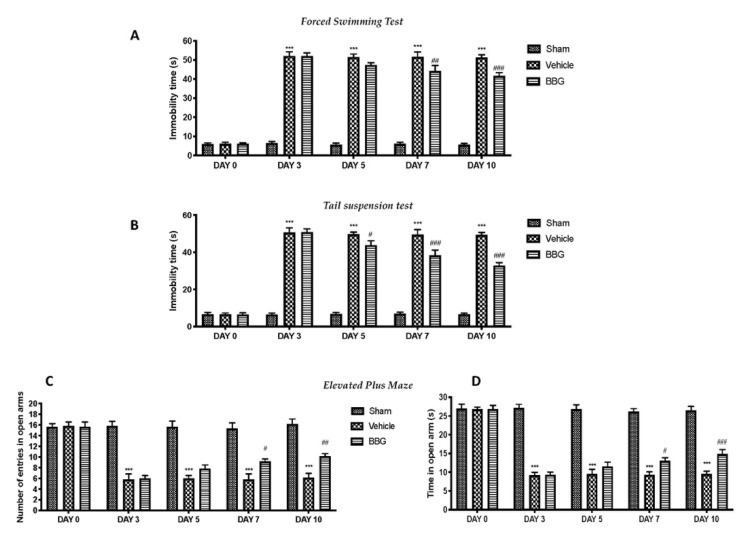
Behavioral analysis: forced swimming test (**A**), tail suspension test (**B**) and elevated plus maze test (time in open arms (**C**) and number of entries (**D**)). Data are representative of at least three independent experiments and are expressed as mean ± SEM from N = 6 rats/group. *** *p* < 0.001 vs. sham; # *p* < 0.05 vs. vehicle; ## *p* < 0.01 vs. vehicle; ### *p* < 0.001 vs. vehicle.

Depression and anxiety behaviors are closely associated with FM. Thus, the depressive-like behavior was evaluated by forced swimming test (FST) and tail suspension test (TST). Seven days after the last reserpine injection, the vehicle animals showed a significant increase in the immobility time, compared to the control group. BBG administrations significantly decreased the duration of immobility in the FST and TST, compared to vehicle rats (Figure 6A,B, respectively).

Additionally, rats were subjected to the elevated plus maze (EPM) test to evaluate post-injury anxiety and risk-taking behaviors. An increase in open arm activity (duration and/or entries) reflects anti-anxiety behavior, as demonstrated in sham animals. Reserpine-treated rats showed a significant decrease in duration (Figure 6C) and entries (Figure 6D) in open arms, while BBG at the dose of 50 mg/kg significantly relieved these anxious–depressive symptoms. 

### 2.7. Effects of Inhibition of P2X7R by BBG on Assessment of Hippocampal Neurogenesis

**Figure 7:**
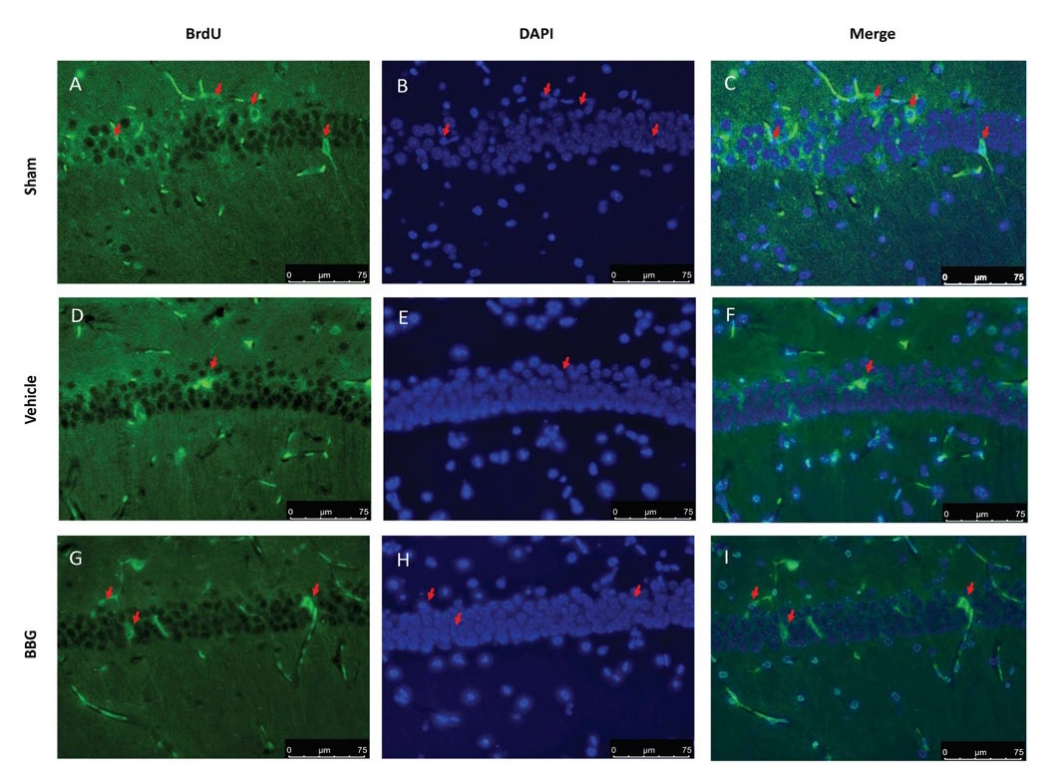
Immunofluorescence evaluation for BrdU expression: sham (**A**–**C**), vehicle (**D**–**F**), BBG (**G**–**I**). The red arrows indicate co-localization of positive cells. Data are representative of at least three independent experiments and are expressed as mean ± SEM from N = 6 rats/group.

Changes in adult hippocampal cell proliferation were assessed by bromodeoxyuridine (BrdU) administration for seven days after last reserpine injection. Immunofluorescence staining showed a significant decrease in the number of BrdU-positive cells in the hippocampal region in vehicle rats (Figure 7D–F), compared to the sham group (Figure 7A–C). Inhibition of P2X7R by BBG administration restored the hippocampal neurogenesis (Figure 7G–I).

## 3. Discussion

Fibromyalgia (FM) is a multifactorial chronic disease that affects up to 6% of the population [1] and is one of the most commonly seen pathological disorders in primary care [27]. The degree of improvement achieved by many drugs prescribed for FM is modest at best; in fact, 40–60% of patients do not respond to drug therapy and most fibromyalgia patients are sensitive to the side effects of these medications [1,28]. Given the substantial reduction in quality of life in patients with FM, elucidating the pathophysiology and developing improved therapeutic approaches are urgent issues, and well-characterized animal models of FM can contribute to resolving them.

In this regard, purinergic signaling is often associated with pain and inflammatory responses [29]. For instance, the purine molecule ATP has been demonstrated to play an important role in regulating nociceptive transmission in different regions of the spinal cord and brain, and promotes the release of mediators involving pain [12,30,31]. Therefore, targeting the specific receptors for ATP (e.g., P2X and P2Y) could be effective in modulating pain conditions [11]. Among the ligand-gated P2X-Rs, P2X7R has been largely studied in different pain states. For a long time, “large pore formation” had been the central mechanism to explain P2X7R actions [32]. However, this receptor is known to have other peculiar actions that distinguish it from other ligand-gated channels. In fact, it has been shown that P2X7R activates some intracellular signal transduction proteins and cellular processes. Several studies suggest that P2X7R is an important, even a central player in key processes related to inflammation, such as the activation of IL-1β processing, most probably via the NLRP3 inflammasome complex [33]. Recent interest in the P2X7R field concentrates mostly around areas of cancer and inflammation [34,35]. ATP has been seen as an important mediator in inflammation, neuroinflammation and tumor biology as its concentration can reach high levels around the cells in an inflamed area or inside a tumor, thus potentially providing a sustained background stimulus regulating and affecting the function of immune and neoplastic cells [32]. P2X7R is a good candidate as a target due its relatively low sensitivity to ATP; additionally, it seems to participate in the development of central sensitization [36]. Several pieces of evidence demonstrated that the absence or inhibition of P2X7R led to the disappearance of mechanical and thermal hypersensitivity in models of neuropathic and inflammatory pain, whereas normal nociceptive processing is retained [37,38]. Therefore, the objective of the present study was to examine whether targeting P2X7R may be regarded as a key upstream factor to block pain signaling and neuroinflammation associated with FM. To do this, we used an experimental model of reserpine-induced FM and the animals were treated with BBG, that due to its high selectivity and low toxicity, make this compound an ideal candidate for blocking the potential adverse effect of P2X7R activation after FM induction.

First, we evaluated whether daily BBG administrations were actually able to reduce P2X7R protein expression in the spinal cord. Our data confirms the antagonistic effect of BBG on P2X7R activation, laying the groundwork for studying the possible protective effect of BBG. Henceforth, subsequent results show the possible mechanisms by which receptor inhibition can exert its positive effects on the management of FM.

Therefore, we have investigated the cellular and molecular mechanisms involved in pain signaling. A growing number of studies have demonstrated the importance of mast cell activation in FM and painful conditions [39]. Mast cells reside near the nerve fibers, which allows them to migrate in order to modulate nociception and neural activity [40]. Experimental animal models have shown that intense or prolonged nociceptive receptor activation can result in a protracted increase in the excitability of dorsal horn neurons, designated as central sensitization, where MCs play an important role. As a consequence of their migration and degranulation, there is a significant release of pro-inflammatory and neuro-sensitizing mediators, such as NGF and c-FOS [41,42,43]. Our results are in line with previous studies, as shown by the high number of intact and degranulated mast cells in the dorsal horn of the spinal cord, seven days after the last reserpine injection. Consequently, an increased expression of pain-related pro-inflammatory mediators was detected in spinal cord tissues after FM induction. Inhibition of P2X7R by BBG decreased mast cell activation and degranulation, alongside the related upregulation of pro-inflammatory cytokines and pain mediators. Not only are mast cell infiltration and degranulation involved in pain signaling, but they also induce glial cell activation and neuroinflammation. In turn, the literature data reported that spinal microglia powerfully regulate pain transmission and intensity [44]. P2X7R appears to play an important role in inflammation and neuroinflammation [34]. This is understandable, considering that P2X7Rs are expressed by immune and inflammatory cells and are upregulated in inflammatory processes, including mast cells, dendritic cells and microglia, the most widely studied cells from the monocyte/macrophage axis [29]. Consistent with the latter notion, we investigated GFAP and Iba-1 expression; astrocytic and microglial activation markers, respectively. Immunohistochemical analysis showed a significant increase in GFAP and Iba-1 expression, following reserpine injections. On the contrary, daily administrations of BBG significantly prevented both astrogliosis and microgliosis in spinal cord tissues. Furthermore, we observed substantial neuroinflammation not only in spinal cord but also in brain regions regulating pain sensation. In particular, it was recently proposed that FM may involve localized inflammation in the hypothalamus [26]. Indeed, some chronic pain conditions, including fibromyalgia, have been directly linked to abnormalities in the hypothalamus–pituitary–adrenal axis (HPA) [45]. In addition, regulation of nociceptive input originates in the hypothalamus and is transmitted to the spinal cord via brain stem nuclei in the medulla [46]. In line with this, our immunohistochemical analysis showed an increase in the number of positive cells for both GFAP and Iba-1 in hypothalamus area, while the inhibition of P2X7R by BBG significantly reduced both markers of neuroinflammation.

Additionally, the role of P2X7R, an ATP-gated transmembrane cation channel, in the signaling cascade has received particular attention due to its extensive involvement as a key regulatory element of NLRP3 inflammasome activation [47]. To date, there are six known inflammasome multiprotein complexes, but the NLRP3 inflammasome is one of the most well studied, being expressed primarily by immune cells such as macrophages and neutrophils, as well as dendritic cells, microglia and dorsal root ganglia to a lesser degree [10,48]. This complex includes NLRP3, a NOD-like receptor that is a sensor for the activation of the inflammasome, an apoptosis-associated speck-like protein containing a CARD complex (ASC), and the serine protease caspase 1 (Casp-1) [49]. The activation of NLRP3 leads to the maturation of Casp-1, which is subsequently implicated in the cleavage of pro-IL-1β and pro-IL-18 into the biologically active cytokines [50]. The release of both cytokines is known to directly or indirectly sensitize nociceptors and to cause pain [51] in several painful inflammatory and non-inflammatory conditions such as rheumatoid arthritis, neuropathic pain and diabetic wound healing [10,52]. Therefore, targeting its ATPase activity by inhibition of P2X7R, the activation of the NLRP3 inflammasome and the consequent release of proinflammatory cytokines are prevented. In line with the above, our study demonstrated that inhibition of P2X7R by BBG reduced the expression of all components of the inflammasome, and reduced proinflammatory cytokine levels, in both spinal cord and brain samples.

As we have pointed out several times, the main feature of FM is pain and hypersensitivity; for this reason, we carried out a series of behavioral tests. In the present study, animals subjected to fibromyalgia showed increased pain sensitivity in mechanical allodynia and thermal hyperalgesia, seven days after the last reserpine injection. Moreover, this enhanced sensibility was coupled with depression symptoms, as indicated by the rat behavior in the FST and TST. In the EPM test, reserpinized rats also showed a reduced exploratory activity, indicating an anxiety-like behavior. This finding is consistent with the fact that anxiety disorders are highly correlated with FM and that the depression-like symptoms in rats increased allodynia and hyperalgesia under the condition of FM [53]. Inhibition of P2X7R, through BBG administrations, was able to reduce the increased pain sensibility and the depression-like behavior. As chronic pain is a complex behavior, associated with abnormal mood and memory, it is expected to involve hippocampal processes [5]. Indeed, the hippocampus, a central component of the limbic system, is a crucial mood-regulating region of the brain, also involved in the processing of nociception [54]. Recent data from human patients with chronic pain and rodent models demonstrate several anomalies in hippocampal function, changes in associated behavior and decreases in adult hippocampal neurogenesis [55,56]. Our results are in line with the literature, as demonstrated by a significant reduction in the expression of BrdU in the hippocampal region. On the other hand, the inhibition of P2X7R induced by BBG treatment restored the neurogenesis in the hippocampus.

In conclusion, our results demonstrate that inhibition of P2X7R with the antagonist BBG modulates mediators involved in pain sensitization, neuroinflammation and NLRP3 inflammasome activation. Therefore, the P2X7R target could represent a possible approach for the development of new drugs in the treatment of fibromyalgia.

## 4. Materials and Methods 

### 4.1. Animals

Sprague–Dawley rats (200–250 g, male; Envigo, Italy) were housed in a controlled environment with free access to typical rodent diet and water. This study was approved by the University of Messina Review Board for the care of animals. Animal care conformed to Italian regulations on the use of animals for experimental and scientific purposes (D.Lgs 2014/26 and EU Directive 2010/63).

### 4.2. Induction of Fibromyalgia

Reserpine administration was performed by subcutaneous injection of 1 mg/kg for three consecutive days [41]. Reserpine (Sigma-Aldrich, Saint Louis, MO, USA) was dissolved in distilled water with 0.5% acetic acid (vehicle). Sham animals received the same volume of vehicle, but no reserpine administrations.

### 4.3. Experimental Groups

Rats were randomly divided into several groups (n = 18 for each):

1. Reserpine + saline: Rats were subjected to injection of reserpine as previously described and treated with saline (intraperitoneally, i.p.) for 7 days starting from the day after the last reserpine injection;
2. Reserpine + BBG: Rats were subjected to injection of reserpine as previously described and treated with BBG (50 mg/kg, i.p.) for 7 days starting after the last reserpine injection;
3. Sham operated groups: Rats were injected subcutaneously with vehicle (distilled water with a final concentration of 0.5% acetic acid) instead of reserpine and treated intraperitoneally with saline or BBG (50 mg/kg) for 7 days starting from the day after last vehicle injection. Since no significant histopathologic or behavioral change was found between sham groups, we present data of sham + saline groups only for analysis.

The dose and route of administration of BBG were chosen based on previous studies [12,57]. At the end of experiment, animals were sacrificed and the L4–L6 area of spinal cord and brain samples were collected and fixed in 10% neutral buffered formalin and embedded in paraffin for histological, immunohistochemical and immunofluorescence examinations, or stored at −80 °C for further analyses. All analysis was carried out at the end of the experiment, i.e., 7 days after the last injection of reserpine (3 days of reserpine injection + 7 days of BBG (or saline) treatment = 10 total days).

### 4.4. Bromodeoxyuridine (BrdU) Treatment

To assess newly generated neurons and proliferated cells in hippocampus, rats received BrdU (50 mg/kg, i.p. dissolved in saline) every day for seven days after last reserpine injection [58]. BrdU incorporation into cell nuclei was assessed by immunohistochemistry.

### 4.5. Staining of Mast Cells

Seven days after last reserpine induction, the lumbar vertebrae with spinal cord (L4–L6) tissues were fixed, cut and stained with toluidine blue for identification of mast cells, as described previously [59,60]. Every section was observed at a magnification of 100×, using a Leica DM6 microscope (Leica Microsystems SpA, Milan, Italy) associated with Leica LAS X Navigator software (Leica Microsystems SpA, Milan, Italy).

### 4.6. Western Blot Analysis 

Western blot analysis was performed on spinal cord and brain samples with the protocol that we previously described [61,62,63]. Specific primary antibody: anti-P2X7R (1:1000, Cell Signaling Technology, Danvers, MA, USA); anti-NGF (1:500, Santa Cruz Biotechnology (SCB), #sc-32300), anti-c-FOS (1:500, SCB, sc-166940), anti-NLRP3 (1:1000, Cell Signaling Technology), anti-ASC (1:1000, SCB, #sc 271054), anti-Casp-1 (1:1000, Cell Signaling Technology) were mixed in 1× PBS, 5% *w*/*v* nonfat dried milk, 0.1% Tween-20, and incubated at 4 °C, overnight. After, blots were incubated with peroxidase-conjugated bovine anti-mouse IgG secondary antibody or peroxidase-conjugated goat anti-rabbit IgG (1:2000, Jackson Immuno Research, West Grove, PA, USA) for 1 h at room temperature. To assess whether blots were loaded with equal volumes of protein lysates, we probed them with a mouse monoclonal β-actin (1:5000; SCB, #sc8432). Signals were detected with an enhanced chemiluminescence detection system reagent according to manufacturer’s instructions (Super-Signal West Pico Chemiluminescent Substrate, Pierce, Altrincham, UK) [64]. Protein expression was quantified by densitometry with BIORAD ChemiDocTM XRS+ software and standardized to β-actin levels. Images of blot signals were imported to analysis software (Image Quant TL, v2003) [65].

### 4.7. Immunohistochemical Analysis

Immunohistochemical analysis was performed on sections (7 μm) of spinal cord and brain, as previously described [66,67,68]. Briefly, after deparaffinization, endogenous peroxidase was quenched with 0.3% H_2_O_2_ in 60% methanol for 30 min. The sections were permeabilized with 0.1% Triton X-100 in phosphate-buffered saline (PBS) for 20 min. Non-specific adsorption was minimized by incubating the section in 2% normal goat serum in PBS for 20 min. Endogenous biotin- or avidin-binding sites were blocked by sequential incubation for 15 min with avidin and biotin. The sections were incubated overnight with primary antibodies: anti-GFAP (1:100, SCB, #sc33673) and anti-IBA-1 (1:250, Thermo Fisher Scientific, Madison, WI, USA) antibodies. Slides were then washed with PBS and incubated with a secondary antibody. Specific labeling was identified with an avidin-biotin peroxidase complex and a biotin-conjugated goat anti-rabbit Immunoglobulin G (IgG) (Vector Labs Inc., Burlingame, CA, USA). To verify antibody-binding specificity, we also incubated some slides with only a primary antibody or secondary antibody; no positive staining was found. Images were collected using a Leica DM6 microscope (Leica Microsystems SpA, Milan, Italy) following a typical procedure [69]. As a general procedure, the digital images were opened in ImageJ, followed by deconvolution using the color deconvolution plug-in. When the IHC profiler plug-in is selected, it automatically plots a histogram profile of the deconvoluted DAB image, and a corresponding scoring log is displayed. The histogram profile corresponds to the positive pixel intensity value obtained from the computer program. The number of positive cells was counted in three sections per animal and presented as the number of positive cells per high-power field [70,71]. 

### 4.8. Immunofluorescence Analysis

Immunofluorescence analysis was performed on sections (7 μm) of brain as previously described [72]. Briefly, after deparaffinization, the sections were boiled in 0.1 M citrate buffer for 1 min. Non-specific adsorption was minimalized by incubating in 2% (*v*/*v*) standard goat serum in PBS for 20 min. Slides were incubated overnight with anti-BrdU (1:100, SCB, #sc-32323) antibodies. Sections were washed with PBS and were incubated with secondary antibody fluorescein (FITC)-conjugated anti-mouse Alexa Fluor-488 antibody (1:2000 *v*/*v*, Molecular Probes, UK) for 1 h at 37 °C. Images were collected using a Leica DM6 microscope (Leica Microsystems SpA, Milan, Italy) following a typical procedure [62]. Each picture was digitalized and analyzed as previously described [54,73].

### 4.9. Enzyme-Linked Immunosorbent Assay (ELISA) 

The supernatant of homogenate of spinal cord and brain tissue was centrifuged and operated [74,75]. The expression of IL-1β and IL-18 were measured using ELISA kits (R&D Systems, Minneapolis, MN, USA) following the manufacturer’s instructions. 

### 4.10. Behavioral Testing

In a separate set of experiments, 18 additional animals for each group were used for behavioral testing. Rats were placed in behavior rooms for 5 min for acclimation 2 days prior to the start of behavioral testing. The behavioral tests were conducted by expert observers blinded to the injury status of rats. Tests are described below.

#### 4.10.1. Von Frey Hair Test

Mechanical allodynia was evaluated by electronic von Frey test on day 0 and 3, 5, 7 and 10 post first injection (BIO-EVF4, Bioseb, Vitrolles, France) [41]. The apparatus consisted of a force transducer fitted with a plastic tip. When pressure is applied to the tip, the force applied is automatically recorded. The tip was applied to the plantar area of the hind paw, and an increasing upward force was exerted until the paw was withdrawn. The withdrawal threshold was expressed as the force, in grams, at which the rat removed the paw [76]. 

#### 4.10.2. Hot Plate Test

The hot plate test was performed on day 0 and 3, 5, 7 and 10 post first injection. The hot-plate latency was evaluated using a metal surface maintained at 53.6 °C (Ugo Basile, VA, Italy). Animals were monitored and the licking of a hind paw was acquired as the end point. All the results are expressed as paw-withdrawal latency (s), and maximal latency accepted was 45 s [77].

#### 4.10.3. Tail-Flick Warm Water Test

Spinal thermal sensitivity was assessed by the tail-flick warm water test, on day 0 and 3, 5, 7 and 10 post first injection. Briefly, the terminal part of the tail (4 cm) of the rat was immersed in 50 ± 0.5 °C warm water until tail withdrawal and the duration of the tail withdrawal reflex was recorded. The cut off time was 10 s to minimize tissue damage to the tail [41]. 

#### 4.10.4. Forced Swimming Test (FST)

The FST was performed on day 0 and 3, 5, 7 and 10 post first injection as previously described [8]. Briefly, animals were individually placed in a plexiglass cylinder filled with 23 ± 2 °C water. The time during which the rats stayed immobile was measured, in sec, during a period of 1 min. The immobility time was defined as a total absence of movement except than that which was needed to keep the head above the water.

#### 4.10.5. Tail Suspension Test (TST) 

The TST was performed on day 0 and 3, 5, 7 and 10 post first injection as previously described [8]. Briefly, rats were suspended 50 cm above the floor by means of adhesive tape, placed 1 cm from the tip of the tail. The sum time of the immobility periods during was measured during a test period of 1 min. Rats were considered immobile only when they hung passively and remained completely motionless.

#### 4.10.6. Elevated Plus Maze (EPM)

The EPM test was performed on day 0 and 3, 5, 7 and 10 post first injection, as described previously [78,79], to measure anxiety-like behavior. The apparatus of the EPM consisted of two open arms and two closed arms (50 × 50 cm). Rats were positioned in the middle of the apparatus and the number of entrances and time spent in the open arms were recorded in a five-minute duration using a video camera installed above the EPM [72]. Anxiety was indicated by a decrease in the proportion of time spent in the open arms and a decrease in the proportion of entries into the open arms [79].

### 4.11. Statistical Evaluation

All values are expressed as mean ± standard error of the mean (SEM) of N observations. The images shown are representative of the last 3 experiments performed on diverse experimental days on tissue sections collected from all animals in each group. For in vivo studies, N represents the number of animals used. The results were analyzed by one-way ANOVA followed by a Bonferroni post-hoc test for multiple comparisons. A P value less than 0.05 was considered significant.

## Data Availability

The data presented in this study are available on request from the corresponding author.
